# COVID-Well Study: Qualitative Evaluation of Supported Wellbeing Centres and Psychological First Aid for Healthcare Workers during the COVID-19 Pandemic

**DOI:** 10.3390/ijerph18073626

**Published:** 2021-03-31

**Authors:** Holly Blake, Alisha Gupta, Mahnoor Javed, Ben Wood, Steph Knowles, Emma Coyne, Joanne Cooper

**Affiliations:** 1 School of Health Sciences, University of Nottingham, Nottingham NG7 2HA, UK; 2 NIHR Nottingham Biomedical Research Centre, Nottingham NG7 2UH, UK; 3 School of Medicine, University of Nottingham, Nottingham NG7 2UH, UK; mzyag11@nottingham.ac.uk (A.G.); mzymj6@exmail.nottingham.ac.uk (M.J.); 4 Nottingham University Hospitals NHS Trust, Nottingham NG7 2UH, UK; ben.wood@nuh.nhs.uk (B.W.); steph.knowles@nuh.nhs.uk (S.K.); emma.coyne@nuh.nhs.uk (E.C.); joanne.cooper3@nuh.nhs.uk (J.C.)

**Keywords:** COVID-19, pandemic, psychological wellbeing, workforce, peer-to-peer support

## Abstract

Supported wellbeing centres were set up in UK hospital trusts as an early intervention aimed at mitigating the psychological impact of COVID-19 on healthcare workers. These provided high quality rest spaces with peer-to-peer psychological support provided by National Health Service (NHS) staff volunteers called ‘wellbeing buddies’, trained in psychological first aid. The aim of the study was to explore the views of centre visitors and operational staff towards this COVID-19 workforce wellbeing provision. Qualitative semi-structured interviews were undertaken with twenty-four (20F, 4M) employees from an acute hospital trust in the UK. Interviews were digitally recorded and transcribed, data were handled and analysed using thematic analysis. Interviews generated 3 over-arching themes, and 13 sub-themes covering ‘exposure and job roles’, ‘emotional impacts of COVID-19 and ‘the wellbeing centres’. Supported wellbeing centres were viewed as critical for the wellbeing of hospital employees during the first surge of COVID-19 in the UK. Wellbeing initiatives require managerial advocacy and must be inclusive. Job-related barriers to work breaks and accessing staff wellbeing provisions should be addressed. High quality rest spaces and access to peer-to-peer support are seen to benefit individuals, teams, organisations and care quality. Training NHS staff in psychological first aid is a useful approach to supporting the wellbeing of the NHS workforce during and beyond the COVID-19 pandemic.

## 1. Introduction

On 11 March 2020, the World Health Organization (WHO) declared the coronavirus disease 2019 (COVID-19) a pandemic as a result of rapid worldwide spread. The negative psychological impact of epidemic/pandemic outbreaks (i.e., SARS, MERS, COVID-19, Ebola, and Influenza A) on healthcare workers (HCWs) has been established with risk of anxiety, stress, depression, occupational burnout and post-traumatic stress disorder (PTSD) [1,2,3,4,5,6,7].

Interventions were mobilized at speed in the early stages of the COVID-19 pandemic to mitigate the psychological impact of the pandemic on healthcare workers. Early interventions include digital approaches (e-package, education and support around psychological wellbeing: [8] ‘Be + Against COVID’ platform, web and app support: [9], stress inoculation with peer support and mental health consultants [10], and self-care and organizational approaches to building resilience [11]. However, reviews of interventions carried out during or after disease epidemics and pandemics that are aimed to support the resilience and mental health of frontline workers [7,12] demonstrate the paucity of evidence-based interventions to date. A systematic review and meta-analysis [12] included workplace interventions (e.g., training, structure and communication), psychological support interventions (e.g., counselling and psychology services), and multifaceted interventions [12]. The COVID-19 pandemic has been long-lasting and the growing mental health burden on healthcare workers is undisputed demonstrating the need to implement supportive interventions.

It has been advocated that training in Psychological First Aid (PFA) may be a helpful strategy to support the mental health of healthcare workers during a pandemic [13]. PFA is a world-wide implemented approach to strengthen capacity for the provision of psychosocial support to people affected by an emergency, disaster, or other adverse event [14]. It does not require prior mental health training [15]. According to guidance for the International Federation of Red Cross and Red Crescent Societies [16], “the basis of psychological first aid is caring about the person in distress and showing empathy. It involves paying attention to reactions, active listening and, if needed, practical assistance, such as problem solving, help to access basic needs or referring to further options for assistance. PFA helps normalize worry and other emotions, PFA also promotes healthy coping and provides feelings of safety, calming, and hope”. PFA for healthcare workers was encouraged during previous pandemics [17], and the use of PFA training has been advocated during the COVID-19 pandemic to support the psychological recovery and functioning of the health and care workforce [18,19,20]. In England, on 15 June 2020, PFA training was made available free of charge to all National Health Service (NHS) employees at the forefront of the national COVID-19 response across the country [21].

In addition to the provision of emotional support, rest breaks have been advocated as an important component of approaches to mitigating the psychological impact of a pandemic [19]. Rest breaks are crucial to reducing burnout [22] and during the COVID-19 pandemic, those healthcare workers rarely or never taking breaks were more likely to experience insomnia, acute or chronic fatigue, emotional exhaustion, psychological distress and PTSD [23]. During the first surge of COVID-19 in the United Kingdom (UK), many NHS hospitals trusts operationalized wellbeing centres in order to provide rest spaces for hospital workers. At one pioneering acute hospital trust in England, supported wellbeing centres were established at two hospital sites in April 2020 [19]. These centres were staffed by pairs of volunteers called ‘wellbeing buddies’ who were trained in PFA and provided emotional support and signposting to healthcare workers accessing these dedicated rest spaces. To our knowledge, this was the first study to demonstrate the uptake and reach of COVID-19 supported wellbeing centres, with a high number of centre visits observed (14,934 facility visits recorded over 17 weeks), and high numbers of frontline workers visiting the centres. The centres were perceived positively by visitors and wellbeing was higher in staff who accessed a wellbeing centre compared with those that did not [19].

The overall purpose of this study was to explore staff and service provider views towards supported wellbeing centres as an early intervention designed to mitigate the psychological impact of the COVID-19 pandemic in an acute hospital setting. A qualitative approach was taken to provide in-depth insight. Our research questions were: (i) What is the psychological impact of COVID-19 on NHS workers? (ii) What are the experiences of those who accessed the facility (‘service users’) as well as those who delivered aspects of the service (‘wellbeing buddies’)? (iii) What are the benefits facilitators, obstacles or barriers to accessing or using the facility? (iv) What recommendations can be made for longer-term sustainability of employee wellbeing initiatives?

## 2. Methods

### 2.1. Study Design

This was a qualitative study with interviews undertaken during the COVID-19 pandemic. The research was reviewed and approved in May 2020 by the University of Nottingham Faculty of Medicine and Health Sciences Research Ethics Committee (FMHS REC ref 16-0520). The study utilizes the consolidated criteria for reporting qualitative research guidelines [24] (see Supplementary file S1). The intervention leadership and delivery team were not involved in the design of the research study, or the analysis of data.

### 2.2. Participants and Setting

Eligible participants were hospital employees from an NHS acute hospital trust in England, all of whom had access to the wellbeing centres. The study aims were achieved by exploring views in two groups: (i) employees; (ii) operational staff (centre managers and wellbeing buddies trained in PFA). Employees refer to centre visitors—the term ‘employees’ is used in this context to refer to paid employees who had visited the wellbeing centres, as well as bank staff and contracted hospital volunteers who were working on either of the two study sites during the pandemic. The employee group included staff from both clinical and non-clinical job roles. Operational staff were NHS employees who had been involved in operationalizing the wellbeing centres; these included staff who managed the centres and their facilities (e.g., opening hours, health and safety guidance, refreshment availability, buddy shift rotas), and wellbeing buddies who delivered peer-to-peer psychological first aid. Operational staff sometimes had dual roles, since a number of operational staff had also completed PFA training and undertaken a minimum of one 4-h shift to work as a buddy in a wellbeing centre.

### 2.3. The Supported Wellbeing Centres

A detailed description of the wellbeing centres and wellbeing buddy role is provided elsewhere [25]. Two wellbeing centres opened on 6 April 2020 and provided relaxing rest spaces for hospital employees during the COVID-19 pandemic. Staff could use the spaces for quiet time out, social conversation, or to access emotional support from a wellbeing buddy. The centres were open daily, and refreshments were available. The rooms were highly accessed, with 14,934 facility visits recorded during a 17-week period April–July 2020. The facilities were staffed by 134 wellbeing buddies, working in pairs on a rota system. Buddies were Trust employees who had experienced a reduction in workload in their main roles during the pandemic due to temporary closures of clinics or services. The buddies volunteered to make a temporary transition to supporting the wellbeing centres during this time, with a level of input that varied from a single 4-h shift to several 4-h shifts per week for the duration of the study. Their main role was to provide peer-to-peer support through active listening and signposting to appropriate services. All buddies were trained in PFA by the Trust’s clinical psychology team who provided ongoing supervision and support.

### 2.4. Procedure

Qualitative data were collected during a six-week period between June and August 2020. The study was publicized via employee mailing lists, social media (NHS Facebook groups and official Twitter sites), and regular departmental mailings and publications. Study communications included a link to an online survey, with findings reported elsewhere [19], and details of how to express interest to take part in an individual interview with a member of the research team. Reminders were sent out weekly for the six weeks, with social media notifications posted daily in the final week before survey closure. Study posters were displayed in the wellbeing centres and on staff wellbeing noticeboards around each of the sites.

Written informed consent was taken from all interview participants. Semi-structured interviews were conducted one-to-one, by telephone or video-conferencing facility (Microsoft Teams) and were audio-recorded. There were no incentives provided for participation. Interviews were informed by semi-structured topic guides (Supplementary file S2) and the questioning explored the emotional impact of the pandemic on participants and their views towards the wellbeing centres and wellbeing buddies (see analytic framework, Section 2.5). The guides were developed using the five-step process outlined by Kallio and colleagues [26] which included: (1) identifying the prerequisites for using semi-structured interviews; (2) retrieving and using previous knowledge; (3) formulating the preliminary semi-structured interview guide; (4) pilot testing the guide; and (5) presenting the complete semi-structured interview guide.

Interviews were conducted by a member of the project team (H.B., B.W., S.K.) and all interviewers were trained in interview techniques, research integrity, research ethics and Good Clinical Practice. The number of participants interviewed was based on the number needed to achieve theoretical data saturation. With each interview conducted, the research team judged whether the data emerging was new and satisfying the research purpose. The researchers deemed no new data to emerge at the 23rd and 24th interview, at which point it was deemed that theoretical saturation had been achieved and recruitment ceased. Digital recordings of the interviews were transcribed verbatim and 100% were cross-checked for accuracy by A.G. and M.J.

### 2.5. Data Analysis

Qualitative data were analyzed following the conventions of framework analysis [27,28], with a combined deductive-inductive approach accommodating both a priori issues and those which emerged from the data [27]. Framework analysis is a hierarchical, matrix-based method developed for applied or policy relevant qualitative research where timescales are limited, and the goals of the research are clearly defined at the outset. Three researchers familiarized themselves with the data and any contextual or reflective notes (A.G., M.J., H.B.). First, taking a deductive approach, two researchers independently coded the transcripts with paper and pen (A.G., M.J.), by reading the transcripts line by line, and applying a paraphrase or label (a ‘code’). Codes were pre-defined to address specific areas of interest to the project. Additionally, open coding was conducted on a small number of transcripts (n = 5) to ensure that important aspects of the data were not missed. The researchers compared and contrasted styles of summarizing in the early stages of the analysis process to ensure consistency within the team [27]. A third researcher (H.B.) then reviewed and agreed on the coding in consultation with a hospital employee (service user) to ensure that one particular perspective did not dominate.

Interview data were mapped onto thematic matrices to allow for exploration to address the research questions. Starting with a deductive approach, an analytic framework was used that pre-selected matrices. For employees, this considered (amongst other things): any emotional or work-related impacts of the COVID-19 pandemic, views towards the wellbeing centres, reason for staff access and any impacts of access including any barriers or facilitators; experiences of contacts with the wellbeing buddies (if any), and views on future sustainability of the service. For operational staff (buddies and centre managers), this considered issues outlined above, and their specific views towards the buddy role including reasons for buddies volunteering for the role, views towards the training and support offered and perceived benefits of the buddy role, to buddies themselves and to hospital staff. Then, taking an inductive approach, the researchers included additional themes generated from the data though open (unrestricted) coding. Higher level codes within each theme were refined by grouping lower level codes found in the data. H.B. generated the analytic framework and led analysis, with two other team members (A.G., M.J.) populating the framework, data interpretation and validating the form and content of the framework. In the report of the findings, verbatim quotes with gender and occupational role in parentheses have been used to represent each theme and subtheme.

## 3. Results

Thirty-one people expressed an interest to participate. Written consent was received from 24 participants who comprised the final sample. Seven participants expressed initial interest but were unavailable for interview during the study period due to job demands. Twenty interviews were undertaken using a video-conferencing platform (audio recording only) and four were undertaken by telephone. Participant characteristics are provided in Table 1. Staff visitors to the centres included paid employees as well as bank staff and contracted hospital volunteers working on the study sites during the pandemic. Service delivery participants were those who were involved in operationalizing the wellbeing centre management and/or buddy rotas. The wellbeing buddies were staff members who were trained in PFA and worked shift(s) in the wellbeing centres. Nine of the interview participants had multiple roles, in that some of those who were operationalizing the services, or acting as a wellbeing buddy, had also accessed the centres as a staff visitor at other times. Participants reported their roles and identified their primary viewpoint (Table 1).

### 3.1. Sample Characteristics

One of the participants reported that they had returned from retirement to assist in the pandemic, and another had been redeployed from one clinical area to another. Half of the participants held a clinical job role whereas the other half were in non-clinical occupations. One participant identified as being from a Black, Asian or Ethnic Minority (BAME) group [29]. Ten participants reported more than one perspective, i.e., if they had been involved in some aspect of service delivery or worked at least one shift as a buddy but had also visited the centre for their own respite or wellbeing at other times. Of the sample of 24, 18 had been a centre visitor, 12 had completed shift(s) as a wellbeing buddy and 5 had been involved in some other aspect of operationalising the service. Participants included 11 frontline healthcare workers (5 nurses, 5 allied health professionals (AHPs), 1 healthcare assistant), 3 managers, 5 administrators, 2 ancillary or maintenance staff and 3 hospital volunteers.

### 3.2. Qualitative Interviews

**Figure 1:**
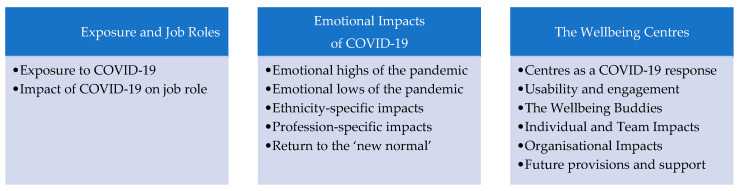
Themes and subthemes.

Interview length ranged from 22 to 63 min and the average duration of interview was 36 min. Interview data analysis generated 3 over-arching themes, with 13 sub-themes (see Figure 1). The analytic framework and codes are provided in Supplementary file S3.

#### 3.2.1. Exposure and Job Roles

##### Exposure to COVID-19

Participants varied in their level of exposure to COVID-19 based on their work or family life situations. For some, this exposure was based on the direct impacts of contact with people who were COVID-positive and the perceived risks of this to their health and that of their colleagues and families. Those who had experienced more direct contact with COVID-19 patients seemed to have experienced greater disruption to their personal lives, and they reported significant impacts on home lives such as changes in caregiving roles, negative impacts on relationships, and periods of complete separation from their families to reduce risk of virus transmission. Most of the participants spoke of overwhelming levels of worry and concern related to COVID-19 exposure, associated with contracting the virus themselves, or the consequences of passing the virus to others. Clinical staff in particular were ‘*nervous about potentially bringing something back to my family*’ (ID106), and highlighted cases where healthcare workers had felt stigmatized within families and local communities, being viewed as a ‘*transmission risk to others*’ (ID135). High levels of anxiety were particularly heightened by ‘*the whole hype around COVID-19 in the media*’ (ID147) and this constant stream of negative information had amplified emotions and staff reported feeling ‘*absolutely terrified*’ (ID118).

Clinical staff had concerns around access to personal protective equipment (PPE), particularly in the early stages of the pandemic, and a lack of access was more commonly reported by those in lower-waged roles. A small number of staff reported feeling inadequately prepared for the pandemic in their clinical roles. This largely related to a perceived inadequacy of knowledge about treatments and protocols for COVID-19, or more generally due to redeployment to new roles, the impact of missed COVID-related training on their ability to work in their usual clinical area, and their personal feelings of preparedness for practice. One healthcare assistant spoke of the challenge of ‘*not being allowed to work in COVID-19 hot zones*’ (ID102) for a period of time due to having missed an essential training session; the participant had been required to work on a non-COVID area but then experienced high anxiety when a number of non-COVID patients developed COVID-19 symptoms and became suspected (but unconfirmed) cases.

Some staff who regularly visited multiple clinical areas in a single day described feeling conflicted when they were unable to complete their usual work and had been restricted to a single area ‘*to stop me potentially spreading the ... virus through the hospital*’ (ID102). Although most of the healthcare workers interviewed reported high anxiety during the pandemic, the dedication of clinical staff was clear. Comments were regularly made about the ‘*challenging but rewarding*’ (ID136) experience of caring for COVID-19 positive patients and the importance of being in a position where they could provide practical and emotional support. This high commitment to the job role was evident as staff reported a desire to be physically present in the hospital setting despite their worries about virus transmission, and they communicated a strong sense of duty and responsibility to the NHS, their colleagues and patients. Yet, those staff who had experienced a period of self-isolation due to COVID-19 exposure reported feelings of guilt, frustration and helplessness. These exposure-related emotional burdens were expressed more strongly by those with managerial responsibilities; senior staff felt they were ‘*meant to be reassuring everybody*’ (ID118) which was perceived to be particularly challenging in the context of escalating concerns about COVID and a high level of competing demands on their time.

##### COVID-19 Impact on Job Role

There was no doubt that COVID-19 had impacted significantly on participants’ job roles and responsibilities although participants reported both negative and positive impacts and this often related to the nature of their job role. Frontline healthcare workers described feeling ‘*overwhelmed*’ (ID118) due to how quickly the environment was changing and their increased workload from ‘*more patients and less staff*’ (ID147) with concomitant impacts of work on their stress levels, and for some, devastating consequences for their home lives: *‘it [pandemic] destroyed my relationship*’ (ID115). Many clinical staff experienced extreme changes in roles and responsibilities if they had been redeployed to work in new and unfamiliar areas and where some had thrived in this context of change, others had struggled with job-related transitions. Those who experienced greater difficulty with changes in the work areas or the transition to new roles were more commonly those who had worked in a single specialty for many years, or those for whom their usual clinical working environment had become fully COVID-focused in a short space of time. For example, one staff member reported that their usual work area was a clinical ward for older people’s care but due to the high number of positive cases, the ward was reassigned to become a COVID-19 positive area.

In addition to changes in clinical areas and job roles, the pandemic facilitated rapid changes in day-to-day operations and the way in which staff communicated. Due to high workload, remote working or social distancing, the transition to online rather than face-to-face meetings was seen by many to be revolutionary, particularly for clinical staff or those in senior roles: ‘*it transformed ... the amount of time that ... we are saving because we are not jumping on the [hospital transport] between sites*’ (ID118). Staff referred to a greater level of efficiency in team communication with the transition to video meetings. Clinical staff spoke of prior ‘*impracticality*’ and ‘*wasted time*’ (ID126) of face-to-face team meetings involving colleagues from different sites and they valued the flexibility and efficiency provided by access to video-conferencing technology. Conversely, some staff struggled with the volume of online meetings, particularly administrative staff who were working from home: ‘*I*’*ve always had a lot of meetings, but it seems even more so now, and the meetings are all back-to-back*’ (ID104).

The pandemic brought with it sense of cohesion and team effort across the Trust to pull together in the fight against COVID and this was irrespective of job role or level of seniority. Some alluded to ‘*stepping up*’ (ID116) in their job roles, for example, hospital volunteers who spoke of a necessary transition from ‘*meet and greet*’ to ‘*more substantial roles*’ (ID120) that would allow them to help support NHS staff by ‘*keeping the hospital safe*’ (ID147). The remit of their role had changed completely: ‘*it was a totally different role because … we were meet and greet beforehand, and it was touch point cleaning, general duties taking non-COVID patients around, dropping letters off or prescriptions off at wards different things like that*’ (ID120).

There was a general concern about the reduced capacity within the NHS for broader treatment and care for non-COVID patients. Many staff shared their worries about high volume of patients avoiding seeking healthcare during the pandemic for fear of contracting COVID-19, and the impact this would have on their work volume and job roles the following year. On a more profound level, staff raised serious concerns about the long-term health and mortality impacts of reduced help-seeking in the general public, alongside the temporary closure of outpatient clinics and services during the pandemic.

#### 3.2.2. Emotional Impact of COVID-19

##### Emotional Highs of the Pandemic

Many participants felt exhausted from the first wave of COVID-19 in the UK and had therefore struggled to articulate an emotional ‘silver lining’ to the pandemic more broadly at the time these interviews were conducted. However, some of the participants referred to feelings of ‘*contribution*’, ‘*usefulness*’, and ‘*reward*’ (ID126) from supporting the NHS, their patients and their colleagues. Some spoke of a noticeable increase in “*camaraderie*” and team spirit and referred to: “*we*’*re all in this together*” (ID123), “*We*’*ve done things you know as a team, we*’*ve done things individually ... but with the aim being for the team*” (ID107). For these participants, there was a consensus that the wellbeing centres had brought significant benefits for mental wellbeing that were perceived to have been ‘*critical*’ to staff ‘*survival of COVID-19*’ (ID132) during this time. These emotional impacts are explored in more detail within the wellbeing centre theme.

##### Emotional Lows of the Pandemic

Several participants reported that this research interview had been their first opportunity to stop and reflect on the shock, and impact of COVID-19 on their own mental wellbeing. Managing death and grief seemed to be the most notable challenge for these healthcare workers. Several of the participants were tearful during the interviews as they reflected on events that had occurred through the first wave of the pandemic. Some of the nurses and allied health professionals that were interviewed had explicitly stated they had not previously spoken about their own feelings because of the high focus on the management of patients and workload through this difficult time. Many participants reflected on the shock of seeing ‘*death after death*’ (ID126), with a rapid and uncontrollable escalation of sickness and death which they perceived to have been ‘*traumatic*’ and ‘*left emotional scars* (ID125)’. Others spoke of frantic shifts managing high numbers of admissions with a shortage of beds for a ‘*tsunami*’ (ID126) of patients, only to return on their next shift to ‘*rows of empty beds*’ (ID136) and ‘*knowing what that means*’ (ID136) but having to block out their own feelings to prepare for the next influx.

The rapid deterioration of patients caused significant worry for staff. This related to patients admitted with COVID-19 as new admissions, and existing patients who were already hospitalized with a serious illness who had contracted COVID-19 during their hospital stay. The rapid decline of existing patients had presented a particular emotional challenge for some staff: ‘*...seeing patients that were progressing quite well ... being impacted by COVID-19 and that completely kinda wiping out their recovery and having to start from scratch. That’s been quite hard*’ (ID147). Several staff expressed despair that they were not able to provide adequate support and care for patients with other serious conditions: “*it was ... devastating, because it’s not that people aren’t having strokes, it’s that they’re not coming in, so they’re dying at home or they’re having strokes and not getting the rehab they need*’ (ID147).

High levels of stress, anxiety and worry had led to physical and emotional exhaustion for both clinical and non-clinical staff and there were frequent reports of ‘*feeling drained*’(ID125), ‘*overwhelmingly tired*’ (ID116) and ‘*being fatigued*’ (ID132) Clinical participants had experienced significant ‘*guilt for not being able to help*’ due to family circumstances or government regulations (e.g., one or more periods of self-isolation). For clinical staff who were working remotely or self-isolating, an inability to make a clinical contribution during the peak of the pandemic had significantly challenged their professional identify and this was experienced deeply, from feelings of ‘*not really doing anything*’ through to perceiving that their career had ‘*gone in a different trajectory*’ (ID118).

The need to manage patients and visitors in rapidly changing circumstances was difficult for staff and volunteers who were challenged to provide support in a changing context and often with limited information due to shifting processes and procedures: The lack of control and emotional labour created a highly stressful environment and at times, helplessness: *“...emotional people coming in who didn*’*t know where to go, what to do, what the protocol was, how things would, would, pan out and how things happen and we were the same you know, it was ever moving*” (ID135).

A recurring theme was the inequity in support for health and wellbeing between different staff groups. Participants working nights or weekends expressed frustration and alluded to being a ‘*forgotten group*’ where wellbeing and mental health support was concerned. A wellbeing buddy spoke with trepidation about the impact of COVID-19 on shift-working frontline care staff: *“there*’*s no two ways about it, there*’*s far fewer people around to provide support. So, I think that that is a shame*’ (ID110). Lower-waged workers and volunteers reported high levels of anxiety and alarm at the lack of PPE for non-clinical staff in the early stages of the pandemic: “*we weren*’*t asked to wear any masks or any PPE whatsoever, the only PPE we actually wore was some gloves and an apron, umm, when we did our touch point cleaning*’ (ID135). Stress levels were high for these participants as they spoke of moments of realization that they had endured so much time in a hospital building without PPE during the early stages of the pandemic, often with high movement around the building, in and out of clinical areas, or greeting visitors to the hospital: ‘*we were greeting these people [COVID-19 patients]*’ (ID135). They described how frightened they had been (and still were) about the consequences for themselves, their patients and their families.

##### Ethnicity-Specific Impacts

High emotional burden was coupled with worry and anxiety resulting from constant media reports about the deaths of NHS staff which impacted on staff morale, and the disproportionate impact of the virus on BAME communities. Participants reported that this ‘*became intensely real*’ as people’s friends, colleagues and family members became sick with COVID-19: “*I’ve a really good friend and colleague who lost their mother of a BAME ethnic background and that was really hard*” (ID145). Healthcare workers had observed or experienced ‘*desperation*’ amongst staff around access to PPE, and arguments between team members over PPE, although they generally interpreted this to be fueled by the heightened emotions at the time, specifically the fear and anxiety associated with perceived COVID-19 risk to themselves and others: “*...when people are not as kind as they could be, that’s because actually they are fearful and they are upset ... and they wanted to have a higher level of PPE than the organization was actually promoting*”(ID101). Some participants alluded to changes in behaviour over time as staff became ‘*more considerate*’ as the higher number of deaths in BAME communities became apparent, and they reflected on the negative impact that this had among colleagues in the NHS workplace. At the outset, awareness of these health disparities had been lower: “*no we weren’t aware that this affected our BAME community as much*” (ID135); and this advanced alongside the pandemic as staff were, “*discussing things around, you know, what it meant for them and ... you could tell the differences*” (ID145).

For a minority, the apparent disproportionate impact of COVID-19 on BAME staff and patients generated a certain level of fear and stigma. A few non-BAME participants admitted that at some point during the pandemic, they had perceived themselves to be at greater risk of contracting COVID-19 from BAME colleagues and patients. There were reports of bi-directional fear among non-BAME staff related to working in clinical areas with high numbers of BAME patients who had been hospitalized due to COVID: *“it added a bit more pressure actually to us I think, when we were dealing with ethnic minorities, because we felt there may be more of a risk to them from us and vice-a-versa from them to us”* (ID135). However, this was recognized to be unhelpful and detrimental to the wellbeing of all involved as it contributed to the spread of fear in the workplace environment. For the vast majority of employees, a growing sense of team cohesiveness had become apparent over time. Many of the participants alluded to staff becoming increasingly protective towards those they perceived to be at greater risk of COVID-19 related hospitalization, including BAME colleagues and staff with underlying health conditions: *“...being aware of who’s on shift and making sure that everybody’s got the correct PPE”* (ID147). As knowledge about COVID-19 risk increased, this was seen to be a catalyst for team-related behavioural change, with colleagues putting their own anxieties aside to volunteer for additional shifts to cover their colleagues and reduce their risk of virus exposure: *“...staff ... would come in, certainly as the media started to talk more about our BAME colleagues being more at risk”* (ID103). Staff recognized the vulnerability and heightened anxieties of BAME staff and teams endeavoured to support BAME colleagues which was viewed as a positive, but participants also noted the negative impact of this, which was increased worry and concern as to whether certain team members could safely engage in their normal work activities.

##### Profession-Specific Impacts

Despite the negative impacts of the pandemic, there were some positive impacts reported relating to redeployments, volunteering roles, changes to job roles, increased use of technology and the opening of the wellbeing centres. These were all seen to have increased opportunities for staff to work collaboratively and to meet other NHS workers. This was unanimously perceived to be beneficial to any professions, providing significant increases in ‘*opportunities for low-level networking*’ (ID129) and a better understanding amongst staff of how COVID-19 had impacted individuals and teams across the whole hospital trust: *‘we could ... just ask them a couple of questions about how it had affected their workload, say in anaesthetics, or accident and emergency doctors*’ (ID129). The impact of COVID-19 more broadly together with an increase in the mixing of different professions had allowed staff from diverse job roles to integrate better, and this was seen to have increased co-operation: *“we had issues with catering staff before this ... before it has been difficult you know to get food out of hours for people ... well they can*’*t do it, for a variety of reasons”; “the minute we sit down and said we were COVID ... they absolutely smashed it ... catering were absolutely amazing throughout COVID for us.*’ (ID123)

Several clinical staff referred to ‘*tensions between teams*’ that existed prior to pandemic, which had been positively influenced by the need to pull together to ensure quality of patient care through the crisis. Conversely, the redistribution of staff members across the trust had impacted negatively on some clinicians who referred to pandemic-related role changes being ‘*very disruptive to the team*’.

Volunteers and lower-waged staff reported that they ‘*felt more of a part of a family*’ and more ‘*included*’ in the workforce ever before. Staff from these job roles were seen by themselves and others to have a pivotal role in the NHS through the pandemic and this appeared to have enhanced self-esteem and sense of value.

##### Return to the New Normal

The rapidity of change during the pandemic was applauded for the most part and staff were wholly positive about the systems and processes that had been put into place to cope with the crisis, including increased efforts to support staff wellbeing. Many had learned new skills, met new colleagues, and taken on new challenges and roles and this had built self-efficacy and confidence and for some, future career opportunities. Staff had felt valued by their employer during the pandemic and wanted to retain this feeling moving forwards. However, almost all staff interviewed had concerns about supportive provisions being reduced in the future when the pandemic subsides, and they used terms such as *‘slipping back*’ and services *‘dropped off*’ when *‘the NHS goes back to more normal operating standards*’ (ID129). Most staff alluded to the impacts of the pandemic on staff mental health as long-term and they were worried about the emergence of more serious psychological problems later down the line once the immediate threat of COVID had subsided: *‘there*’*s gonna be a lot of, delayed stress, guilt, mental health impact, because people have been in survival mode for crisis*’ (ID129). This was a concern for most participants, and there was a consensus that staff had not had time to reflect on the situation during the crisis, and that when they did, services would need to be increased, not reduced: *‘people are going to start feeling and processing their experiences, which they*’*re probably going to need support with*’. (ID129)

Beyond the psychological support required during and post-pandemic, many staff spoke very negatively about the hierarchical nature of the NHS (during normal times) and they described life during the pandemic as to some extent, ‘*professionally free*’. Subsequently, concerns were raised that the feelings of ‘*togetherness*’ experienced by hospital staff during the pandemic would slowly fade, and that the NHS and their employing organization would return to more segregated operations and participants believed that this would hinder progression and lower staff morale. As discussed previously, there was a strong consensus that ‘*integration*’ enriched the work environment and was better than their usual experience of ‘*separation*’, not least for productivity and performance, but also for morale and staff wellbeing.

#### 3.2.3. The Wellbeing Centres

##### Centres as a Workplace COVID-19 Response

The centres were viewed as an essential support strategy for staff during this time. All the staff that were interviewed spoke about the wellbeing centres in a positive light and in relation to this, referred to the trust as ‘*responsive*’, ‘*supportive*’ and ‘*valuing its staff during difficult times*’ (ID110).

Many participants spoke with pride about the ability of NHS teams to come together during difficult times, in order to implement organizational changes that were perceived to be both appropriate, and necessary. Participants expressed a pride to be part of the NHS, and as such, a member of a capable workforce that was able to respond at speed in a crisis situation. The wellbeing centres were viewed to be part of this pandemic response, and interventions to support staff were deemed to play a critical role in ensuring that the workforce was able not only to function but thrive during the COVID-19 pandemic. Staff spoke highly of the ‘*agility*’ of leaders in mobilizing this initiative in a short timescale, and in general, the organizational leadership team were applauded for the rapid establishment of what was seen to be a critical component of the COVID-19 response. Participants spoke about their confidence in the NHS workforce who had, during the pandemic, acted quickly and taken control, pulling teams together and initiating change to respond to the crisis. They emphasized the capability of the NHS workforce and how this capability was accentuated in a crisis situation. However, in sharing these views, participants expressed frustration with the ‘*red tape*’ that existed within the organization and across the NHS more broadly, that, ‘*during normal times*’, hindered progress, slowed, or prevented innovation and the implementation of new initiatives in general, irrespective of the focus on staff wellbeing, or patient care.

There was a prevailing view that the investment of resources in staff wellbeing had been (and remained) essential to sustaining the capability of the workforce during the pandemic. Participants recognized that their employer had significantly invested in staff wellbeing services prior to the pandemic. The value of existing interventions to the non-clinical workforce was clearly recognized, but it was frequently raised that existing services were primarily centred around the promotion of healthy lifestyles (such as diet and exercise) and did not adequately address some of the core issues that impacted on the wellbeing of frontline care staff. Although participants were positive about workplace health promotion, few of the staff interviewed were regular users of the existing staff wellbeing services, which were referred to with language such as ‘*nice to have*’ or ‘*perhaps most appropriate for office staff*’ (ID110). Particularly for clinical participants, the COVID-19 wellbeing centres were seen to be ‘*critical*’ and ‘*essential*’ (ID132) to address this unmet need, not least during the pandemic but in ‘*normal times*’. Several staff indicated that wellbeing centres should be in place as a standard provision at all hospital trusts. Participants alluded to similar provisions in other hospital trusts that were often called ‘wobble rooms’, although one staff member was critical of this label due to the negative connotations of the word ‘wobble’ (i.e., potentially implying a lack of ability of NHS staff to cope in a crisis). Therefore, participants appreciated the term ‘*wellbeing centre*’ although there was a common view that the purpose of the room could have been better defined in terms of who was able to access it, and the type of support available since this had not been clear to all staff. There was a consensus among participants that this hospital trust had acted appropriately in setting up the wellbeing centres as a COVID-19 response. Yet, some staff expressed concern that these valued support mechanisms would be temporary, pandemic-related measures. It was strongly advocated that wellbeing centres, or other comparable provisions of high-quality rest spaces and psychological support, will be required in the long-term.

##### Usability and Engagement

All of the staff participants had accessed the centres at least once, and the majority were regular users. In terms of accessibility of the centres, views were varied. Those who worked in areas proximate to the centres perceived them to be easy to access and in a convenient location. Others reported that it was time-consuming to visit the centres during breaks due to their location; this view was more commonly held by those with clinical roles who discussed the time impact of leaving clinical areas for breaks, long walks through the hospital site to get there, and having to remove PPE before they entered which resulted in some staff being hesitant to take work breaks. Some staff reported that the opening hours of the centres excluded those who worked night shifts, although there was a recognition of the potential challenges of staffing rooms with support workers if they were open 24-h. There were also barriers to access for staff who were only able to take very short breaks.

“So usually my breaks weren’t longer than half an hour and by the time you’d finished task, got off the ward, you know, taken off your PPE, washed your hands, made your way to the wellbeing centre, you weren’t able to actually be there for a very long period of time before coming back, so if I was, had been particularly run of my feet in the lead up to my break, sometimes it was easier just to sit on the chairs in our normal staffroom on the ward, because that would be a longer period of rest than it was to move over to the wellbeing centre.”

(ID129)

Visitors accessed the centres alone or with colleagues, depending on whether they wanted quiet time out, or social contact. A small number of participants were hesitant to enter the centres alone and reported that this was due either to a lack of confidence, or an uncertainty (particularly for those in non-clinical roles) about who was ‘allowed’ access. Although the centres were open to all, there had been issues with communication since some staff believed that they were targeted to medical and nursing staff, to the exclusion of those in non-clinical roles. Worryingly, some staff in lower-waged and non-clinical roles appeared to feel undeserving of attendance compared with staff in patient-facing roles. Those staff in non-clinical roles had only attended when clinical colleagues had encouraged them, demonstrating the value of supportive teams for the promotion of staff wellbeing.

“As soon as you walk in, the volunteers rushing you a drink and a chat if you need it and, and any support. And yeah, it’s really nice and I think it’s nice to encourage each other as a team and to remember that the wellbeing centers are there, and that they are there for--to use them--if you need them.”

(ID147)

The wellbeing centres were perceived to be a welcoming environment and an ‘*oasis of calm*’. Many of the participants referred to the centres as ‘*a safe space*’ (ID132). Some of the participants were using the centres as an opportunity to engage in small group debriefs about the occurrences of the day. This allowed issues and concerns to be raised and managed away from clinical areas and the immediate pressure of COVID-19. While this practice was observed by other centre users, it was not seen to be disturbing to other visitors, and it was generally accepted that team discussions and off-loading of emotions amongst colleagues was an essential process for clinical staff.

“I used the wellbeing centre because I did a COVID debrief with all of them individually took them off the ward to the wellbeing centre and just sat down and had 20 min half an hour with them after we’d come out or gone into this new pathway just to say ‘how are you?’ ‘how did you find it?’, ‘what could we learn’ ‘what could we have done differently?”

(ID123)

*“…we saw lots of nurses, doctors, front line staff coming in and just really just sitting for 15, 20 min having a coffee, getting away from…the imminent danger and the imminent pressure from what they’re actually doing on the front line.”*

(ID135)

One staff interviewee raised concerns about the risk of confidentiality breaches if clinical information was discussed in a public area and they highlighted that this needed to be reiterated to centre visitors. Generally, there was a consensus that both centres had been highly accessed, without any negative impacts on staff enjoyment of what they perceived to be peaceful surroundings. However, several participants shared that, in the first week weeks of wellbeing centre launch, the volume of visitors was too high. They discussed the availability of donations of food, care packages and gifts that had been provided by local organisations and the general public, as a show of thanks for their efforts during the pandemic. It was generally perceived that while the donations were very well received, the wellbeing centres were not the right avenue for their distribution since a high number of visitors were attending to access gifts without any intention of using the facilities: “*a large number of staff coming in for the wrong reason”* (ID120). Nevertheless, this appeared to be a short-term issue since participants reported that this was resolved within the first few weeks of opening.

##### The Wellbeing Buddies

Due to the pandemic, many of the staff had been redeployed or had temporarily transitioned into different areas of work, and due to the cancellation of outpatient clinics or services, some staff members had experienced significant reductions to their workload during the pandemic. Many of the participants in this situation had struggled with the concept of stepping back from their usual face-to-face commitments to work remotely, as they wanted to be present to offer help and support to their colleagues in the NHS. The opportunity to volunteer to work in the wellbeing centre as a wellbeing buddy was unanimously perceived to be a mechanism by which these staff could assist with ‘*contributing to the efforts of the NHS through the pandemic*’ (ID109). The majority of the buddies interviewed alluded to feelings of value and self-esteem from taking the role; for some, this was a replacement activity for a clinical role that had been put on hold during the pandemic (e.g., due to cancellation of outpatient clinics), for others, particularly those who came to the buddy role from non-clinical positions, this was referred to as an opportunity to contribute to the national and local effort and ‘*step-into a front facing role*’.

“…being able to be what I would call useful at a time of crisis when I am not a frontline member of staff and I’m not clinical. Erm, but that helped me to see that at work I was making a difference.”

(ID101)

Irrespective of the role from which buddies had been redeployed, it was perceived that the provision of staff support was just as important during the pandemic as the provision of patient care. It was widely recognized that patient care cannot be provided without a well-supported workforce. Through operating in newly formed teams, the buddies described this opportunity to change roles and experience something new, but also getting to know other staff in the trust (both buddies, and staff visitors to the centres) and in doing so, gain insight into other departments within the trust and other people’s working lives. It was recognized that a lack of understanding of the role of others could sometimes be a barrier to effective working, particularly between clinical and non-clinical staff. Some buddies spoke of the new teams that had been established during the redeployment had facilitated connections between department and staff, and as such, impacted positively on workplace culture.

*“…making connections with more people across the hospital and understanding, um, other people’s roles because I, I think that is always beneficial, because people work in silence and never understand what people do.”*

(ID101)

*“my fellow buddies were people that I didn’t know, they weren’t from my own service, so it gave me an opportunity to meet some other people and to meet some frontline staff.”*

(ID104)

The training in PFA was viewed as a useful professional development opportunity by recipients. Participants who had undertaken the training described the attainment of transferable skills that would be of benefit in any job role as well as in their home lives during and beyond the pandemic. This training had been provided by the hospital trust clinical psychology team and participants valued the ongoing support that had been offered by this team.

*“The training was really useful and I think everybody should have to go on it, I just think it helps people see things. The training was very simple … it was just nice, insightful, easy to understand. A lot of people are so busy that that they don’t take the time to just sit and watch and listen … the training … it just made you see different things.”*

(ID105)

The staff greatly appreciated the wellbeing buddies and described them as ‘*friendly*’, ‘*supportive*’ and ‘*good at listening*’ allowing staff to offload and share their worries and concerns. The buddies were commended by staff for their knowledge of local and national services. Some staff admitted to needing support but feeling reluctant to ask for it and being directly approached in the wellbeing centre by a buddy facilitated conversations with those who were less likely to seek out help and support. The primary role of the buddies was active listening, and this was the most valued element of their presence in the centres.

*“I think most importantly they’re just someone to listen, they can’t fix all the problems and everything like that, but, they’re people to listen and offload on to and you know, problem shared, problem halved.”*

(ID129)

Some staff were reluctant to talk about their feelings and what they had been experiencing during the pandemic not only at work but also with friends and family. For some, the wellbeing centres (and talking to the buddies) helped them to understand that they were not alone, and that other people were experiencing similar feelings to them. Participants spoke of the high stress and anxiety associated with COVID-19 and the impact of the wellbeing buddies in helping to alleviate this and talk through coping strategies.

*“Well I’ve used them a lot, erm, because I was obviously going through a lot of, you know, stuff in my head…and I found them [centres] very relaxing. To go in there and just be able to just talk a few things through … it certainly gave, err, people a bit of tranquility.”*

(ID115)

For others, the buddies were perceived as a welcome social contact, which one buddy described as: “a *little chat of ‘how*’*s it going and can I get you a drink?*’ *and you know that kind of chit-chat”* (ID104). However, it was evident that even this type of contact was reassuring to staff and engendered feelings of being ‘cared about’ during an emotionally distressing time. Working in this role had positive impacts on the mental wellbeing of the buddies themselves, through the contacts they gained from working alongside other buddies and a sense of purpose they experienced from the role itself: *“…for my mental wellbeing it was just good, it gave me a reason to go out.”* (ID104)

The buddies made good use of resources that were available in the wellbeing centres to facilitate conversations with staff and signposted them to various support mechanisms, such as health and wellbeing apps, counselling, local employee assistance programme and national telephone helplines, occupational health, human resources and their general practitioners. Several buddies spoke of staff ‘*following up on advice*’ or ‘*coming back to give thanks*’, as indicated through subsequent discussions with repeat visitors to the centres and many staff alluded to indirect benefits of conversations with buddies on their wellbeing, personal outlook or team relations on return to work.

##### Individual and Team Impacts

The availability of the wellbeing centres had clear impacts for the psychological wellbeing of staff during the COVID-19 pandemic. The majority of the staff talked about high levels of stress and worry about their own health and that of their families—some clinical staff had moved out of their family homes to protect their families while they were working in certain clinical areas, such as emergency care, intensive care units, or COVID-19 positive wards. Some of the participants had witnessed a high number of deaths during this time, and experienced feelings of guilt and moral distress arising from an inability to provide care in the way they would have preferred. This was due to limited capacity and resources, a high volume of patients and an influx of new hospital admissions, coupled with the uncertainty that existed at the time around approaches to COVID-19 management.

Several of the participants were emotional during the interviews as they spoke of the anxiety they had experienced around the lack of availability of PPE, particularly earlier on in the pandemic, as well as the challenges of balancing work with their home lives (e.g., caring for children or older relatives, worries about accessing food supplies). These emotional impacts of COVID-19 were not just expressed by clinical staff; hospital volunteers spoke of the challenges of working ‘on the front line’ in ‘meet and greet’ roles and the stressors of having daily exposure to members of the public. Ancillary and maintenance workers described concerns about high exposure to COVID-19 from constant movement around the hospital wards during their shifts. One employee with portering responsibilities described the shock of spending full shifts, one after another, transporting the bodies of patients who had died from COVID-19 from their hospital beds to the hospital morgue.

This emotional labour was set in the context of staff from all occupational groups needing time out and needing somewhere to go away from their areas of work where they could regain a sense of normality, even if only for a short time period. It was recognised that some staff needed social contacts and support, whereas others needed to be alone.

“the lighting was fantastic, they had calming music in there, you had some individual booths where if you wanted your solitude you could go into sort of a couple of booth areas where you could just sit by yourself if you wanted to.”

(ID135)

Although some participants spoke of the centres providing a ‘*sanctuary*’ for quiet time, rest and recuperation, others valued the opportunity the centres had provided for social contact whether that was with their team members, or through meeting new people. It was reported that seeing and talking to others increased camaraderie among staff and generated a sense of being ‘*all in it together*’.

“I actually found it quite nice to talk to people in there…you got to hear a lot of people how they were coping with things, and…it did make me feel a little bit better with what was going on in my life thinking I’m not really alone.”

(ID115)

Participants referred to the ‘*relaxing environment*’ and ‘*calming ambience*’ of the centres, which was seen to be important in helping them to psychologically detach from the realities of COVID-19 during their work breaks. Having this rest space was seen to be an essential part of managing individual stress levels, and several participants talked about arriving to the rooms in a highly stressed state but leaving much calmer, able to face the situations they were dealing with and engage with their responsibilities and with others in a more considered way.

As well as providing much needed time out, nursing staff in particular spoke of the overwhelmingly hot working conditions wearing full PPE and they were grateful for the opportunity to be away from clinical areas, have periods without the physical discomfort of PPE that they were experiencing during the working day.

“When we were boiling in our own blood walking around the place, the wellbeing centre seemed to be just this cool, gently lit, quiet, calm corner that you could just step into and as soon as you walked through the door you felt yourself relax, you felt your shoulders go down, you felt yourself breathe easier and there you’d have these lovely people saying ‘hi how are you? can we get you anything? have a seat we’ll bring your tea to you.’”

(ID132)

Many of the staff talked about the difficulties of attending to their own physical needs while working and some reported regularly missing meals and drinks either because they were too busy, or to avoid having to remove their PPE to leave the clinical areas. This led to staff feeling dehydrated and fatigued and some reported they would go full days without going to the toilet. The wellbeing centres acted as a conduit for preventing escalation of the impact of stressors and provided a place where staff could engage in self-care.

*“I would have got more upset if I hadn’t gone into the wellbeing centre. So being able to go onto that ward and you know, go-go back to somewhere, even though it wasn’t really anything to do with COVID. Being able to go back and face going in without immediately bursting into tears which is, I think probably what I would have done had I not had the chance go into the- into the wellbeing centre.”*

(ID118)

“Absolutely essential… just life-savers, literally, um giving us space to sit down and the ability to replenish on fluids and sometimes food.”

(ID131)

##### Organizational Impacts

Participants in the study spoke very positively about their employer and referred to the steps the hospital trust had taken to manage the impacts of the COVID-19 pandemic and support their staff through this difficult time.

“I’m really impressed with the Trust full stop, particularly because of COVID and how they’ve reacted.”

(ID118)

A prevailing theme was that of feeling valued and cared for, although this was juxtaposed with a sense for some staff that feeling valued was ‘*out of the norm*’. One participant alluded to the wellbeing centres as tangible evidence of trust investment in its workforce, and a sign from the organization that ‘*staff matter*’ during the pandemic. However, the same participant proclaimed that this investment in staff during COVID-19 should not be a ‘pandemic response’, but should be retained as a longer-term commitment to staff wellbeing, due to the ongoing pressures on hospital employees and the rising rates of mental ill-health, sickness absenteeism and presenteeism within the healthcare workforce. Staff wellbeing initiatives were believed to incur cost savings for the Trust. Participants shared a view that the wellbeing centres had helped to prevent sickness absenteeism in the workforce during the pandemic due to the impacts on mental wellbeing and reducing stress and anxiety, and this was seen to be even more likely for those who had existing mental health concerns.

“Told them [buddy] about obviously how I was feeling and how mentally I was absolutely drained ... what was going on and ... it was nice to chat I have to admit.”

(ID115)

“We’ve had staff in the past that are still stuck with ... work-related stress and I think this really helps support those people.”

(ID147)

Several of the participants highlighted the impact that the wellbeing centres had on the social cohesion of their team. For some this was due to the individual stress-relieving influence of the centres (facilitated through work breaks, rest and relaxation) which left them feeling energized and rested, and more able to engage with colleagues in a more tolerant and productive way. For others, this was related to the social process of attending centres in small groups and bonding as a team through the process of shared breaks away from their place of work. The relevance of this to the organization was evident in discussions relating to the knock-on effect on their personal and professional relationships and the impact of better team relationships on productivity. Some clinical staff talked about potential impact of taking time out in the wellbeing rooms on care quality, due to the physical and psychological benefits of attending the centres equipping staff to be more compassionate and tolerant towards colleagues and patients, with a reduced risk of errors as a result of work-related stress or fatigue.

*“Makes a team work better together and is more cohesive and is better understanding I think surely that’s got to be safer and beneficial to patient care.”*

(ID131)

However, it was advocated that wellbeing initiatives would only be impactful either with relation to staff wellbeing or to organizational outcomes if staff had the full support of senior leaders within the trust, as champions of staff wellbeing. It was recognized that this support had to come from the most senior leaders, through to managers of teams on a more local level, for staff to feel sanctioned to attend the wellbeing centres (or any other staff wellbeing initiatives at the trust). On the one hand, there was a consensus among those interviewed they had been well-supported by their own teams and line managers who valued wellbeing initiatives and were supportive of staff attending the centres.

“My team leader has been excellent, he said to me, look whenever you’re feeling you need a bit of a pick me up then go there, so he’s been really good. I’ve been quite lucky because my team leader has been very supportive.”

(ID115)

Conversely, there was a general recognition that not all staff were in the same position; participants believed that, for some, staff wellbeing was not well-understood or seen to be a priority by managers. Participants referred to the negative impact this can have across a whole team with wider reaching impacts on staff performance, productivity and quality of patient care. Participants suggested that some managers created barriers to staff accessing the trust wellbeing services, and some suggested that a lack of knowledge (or understanding) about staff wellbeing amongst managers has a significant influence on ‘*how we do the job*’.

“There has to be, um, senior leadership always, because …if you have got a senior leader who is not invested and …quite cynical about it [wellbeing]… ”

(ID118)

##### Future Provisions and Support

As described previously, there was a consensus among staff that the centres had been an essential COVID-19 response, but that the centres were required on an ongoing basis and not just as a temporary pandemic response. It was seen to be vital that staff wellbeing remained a priority all the time. All of the participants felt that there should be established centres or other high-quality rest areas for staff to take work breaks, and that this was not only beneficial, but vital to staff wellbeing. It was strongly advocated that a wellbeing centre or break room should not be within people’s areas of work (a view advocated in particular by clinical staff), and should not be located in an area within the hospital that already had a different, dedicated purpose (e.g., a staff restaurant). There was a shared view that promotion of the centres had not been equal across staff groups, with greater promotion among onsite staff with clinical roles. This had caused some confusion relating to whether staff from non-clinical roles, or staff employed by other organizations but based on or visiting the sites, were able to access the centres and indeed other staff wellbeing provisions. Similarly, it was proposed that more services needed to be available to meet the needs of healthcare staff working rotas, long shifts and night shifts. Participants advocated that staff wellbeing leaders should ensure the needs of all occupational groups are met and provide more clarity around the nature of the services offered and to whom they were targeted. This was deemed to be essential to reduce barriers to service access and for a wellbeing initiative to be seen as inclusive.

All the participants were positive about the availability of wellbeing buddies during the pandemic, and many of the staff participants reported that this role was important and should be considered in the future as a way to support the NHS workforce. However, the buddies themselves highlighted that the role itself may not be sustainable in the longer term as buddies returned fully to their normal job roles.

*“The problem we have got is that because they have all people whose main role was ramped down when the pandemic started, we’re going to start losing them to their main job.”*

(ID120)

The service model during the first surge of the pandemic was therefore seen to be unsustainable in the longer-term as it was unclear how the buddies would be operationalized in the future. However, there was high value placed on the wellbeing buddies and a desire to retain the broader approach of peer-to-peer psychological support. Further, several buddy participants described additional benefits of the role, such as personal and career development opportunities. Some participants had proposed that the role could be undertaken by volunteers with appropriate training and support, although this was not universally supported.

*“You’d definitely want someone that had … even beginning [level] person-centred sort of skills in counselling so that they knew how to, you know, let someone talk and how to actively listen …and then on top of that if you could have some ... erm ... HR or-or some sort of other personnel skills that would be sort of ideal I think... You can ask people to volunteer, erm but you obviously then aren’t guaranteed the quality and the skillset that you’re really looking for.”*

(ID131)

Whilst all of the participants commended the rapidity of the wellbeing centre set up and launch, it was suggested that ‘*some mistakes were made*’ in the early weeks after launch and these needed to be reflected on for future delivery. Participants proposed that, at the outset, the rooms were not necessarily used for their intended purpose, and the availability of donations had created queues to enter, and a higher volume of visitors than was manageable to sustain the COVID-19 social distancing requirement (2m distance between people). It was strongly proposed that the wellbeing centres should be focused only on wellbeing, and that donations for staff needed to be made available in a separate area.

“Some mistakes about accepting donations because that meant that there were people, you know, tens of people queuing to get whatever it was, the freebee that had been delivered that day.”

(ID101)

The concept of providing multiple rooms for different purposes was discussed; participants had observed that some staff wanted to use the centre for ‘quiet time’ or socializing only and did not wish to engage in conversation with buddies, whereas others wanted to offload and share personal information, and this may have been easier in a separate, more private area.

“There was just people who … were happy with someone just making them a drink, err, you know, maybe just have some chit chat … they did have people who had a bit of a wobble and what have you … I’m not sure whether the two should be combined or whether they should be separate.”

(ID104)

There was uncertainty related to whether there were plans in place for continuation of the facilities and staff wanted future plans to be more clearly communicated to them. They also noted that the retention of the centres would require investment of funds post-COVID and it was unclear to participants (either staff or buddies) whether the Trust would be supporting this, although the necessity of ongoing wellbeing support was seen to be paramount.

“Going to need to increase the funding so that they [the trust] can meet the needs of the staff …because I think we’re gonna’ experience more requests than they’re used to, and that might over-run them, because if people can receive support early and get that early intervention, they may need less help in the long run, and be able to stay in work, recover quicker, etcetera. So we don’t want people trying to access support but feeling like they can’t get it.”

(ID129)

## 4. Discussion

The overall purpose of this study was to explore staff and service provider views towards supported wellbeing centres as an early intervention designed to mitigate the psychological impact of the COVID-19 pandemic in an acute hospital setting. Our study highlights the widespread psychological impacts of COVID-19 and the perceived value of high-quality rest spaces and peer-to-peer emotional support.

### 4.1. Exposure and Job Roles

COVID-19 exposure generated fear in our healthcare workers due to perceived virus transmission risk between themselves, friends, family, partners and peers. Fear of being infected and infecting others has been commonplace during COVID-19 [30,31] and other pandemics (e.g., SARS [32]). These fears expressed during the first surge of COVID-19 in the UK were not unfounded [33,34,35] and subsequent research shows that during the first three months of the pandemic, healthcare workers in patient-facing roles were three times more likely to be admitted to hospital with COVID-19 than non-patient facing healthcare workers [36]. Risk was doubled for their household members [36]. Our participants reported concerns about access to PPE, and this stems from national PPE shortages [37], with similar situations reported in the American Nurses Association survey of 20,000 nurses conducted 24 July–14 August 2020 [38].Various concerns about PPE have continued throughout the pandemic, and in January 2021 the British Medical Association has warned about ill-fitting and inadequate PPE and called for enhanced and more appropriate PPE to be made available to staff in all healthcare settings [39]. In addition to PPE concerns, our participants reported negative impacts of changes in workload, work patterns and responsibilities and these factors have all been identified elsewhere and associated with work-related burnout in healthcare workers [40]. Due to COVID-19, there has been a rapid implementation of digital tools and this was broadly viewed positively as a time-saving mechanism to assist communication between colleagues and the continuation of service delivery when face-to-face contact was not possible, albeit with a number of caveats and challenges. The approach to using digital tools in healthcare is undergoing a substantial and rapid shift with potential benefits for patients and employees alike [19,41,42].

### 4.2. Emotional Impacts of COVID-19

In this study, we illustrate the immediate and significant psychological impacts of COVID-19 for healthcare workers from the outset of the pandemic. Our participants reported widespread psychological impacts of the pandemic. They reported experiencing high stress, fear (from perceived vulnerability and media reports on COVID-19 escalation), anxiety and anticipated risk of burnout and trauma; these emotional impacts have been evidenced elsewhere [1,2,3,4,5,6,7]. 

The impact of death and grief on healthcare workers through the pandemic has been significant, and the long-term consequences of this such as burnout and trauma has been recognized. For example, a current study focused on the prevention of burnout in intensive care units (ICUs) will deliver psychotherapist led debriefs to ICU workers, modelled on Death Cafés, which are informal discussions focusing on death, dying, loss, grief, and illness [43]. Moral injury and guilt experienced by healthcare workers has been recently described as an ‘invisible epidemic’ in healthcare workers caring for COVID-19 patients [44]. Moral injury has been defined as “failing to prevent or bearing witness to acts that transgress deeply held moral beliefs and expectations” [45]. The term is often referred to in the context of military service, but has relevance to the experiences of healthcare workers, particularly those working in emergency or critical care services during periods of high stress, such as a pandemic [46]. In such situations, healthcare workers may ethically know the appropriate course of action but be unable to enact this due to organizational or resource constraints, and this not only has implications for healthcare workers’ mental health, but impacts on staff retention [47]. 

It has been advocated that team leaders should help staff make sense of the morally challenging decisions being made [48], encouraging frank discussions about the realities of a pandemic, reinforcing teams and regularly meeting to discuss decisions [48]. One example is a ‘Schwatrz Round’, which provides a forum for staff to safely and openly discuss emotional and social challenges of caring for patients [49]. The evidence also shows that healthcare workers have experienced (or fear experiencing) social stigma and discrimination as a result of their exposure to those affected by COVID-19 [37,50,51] which contributes to psychological distress [2]. Healthcare workers caring for people with COVID-19 have commonly experienced discrimination, alongside people who have recovered from COVID-19, those in lower socioeconomic groups and those with particular religious or racial identities [52]. Rates of presenteeism (being at work in ill-health) are high in healthcare workers [19] and one of the main drivers of presenteeism is mental ill-health [53]. The emotional impacts of the COVID-19 pandemic are therefore likely to have significant financial and resource implications for organizations since the economic cost of presenteeism far outweighs that of absenteeism [54,55]. All these factors indicate that the emotional impacts of the pandemic were significant, and that staff had made personal sacrifices during this time. With the emergence of burnout and signs of trauma, it is clear that psychological support will need to be available for healthcare workers for the longer-term.

Healthcare workers’ belief that they were ‘making a contribution’ during the pandemic seemed to be important for their emotional wellbeing and to some, their sense of professional identity. This aligns with prior research highlighting the solidarity between colleagues during the pandemic, and feelings of being valued by society [56]. The sense of contribution experienced by our participants appeared to be irrespective of whether they worked in a clinical or non-clinical role, or were physically present in the hospital setting during the first wave of the pandemic (e.g., some participants were remote working during this time, others had been shielding if they were clinically vulnerable, or had self-isolated at some point during the pandemic due to COVID-19 exposure). This has been recognized by hospital trusts and some employers provided guidance or recommendations for how to help staff who were unable to engage in their normal roles to contribute to the national effort, for example through non-clinical activities, and remote support [53]. However, it has been found that some healthcare workers unable to work on the frontline during the pandemic not only felt guilt, but perceived themselves to be undervalued, which demonstrates the importance of colleagues and managers in healthcare organizations proactively acknowledging their contribution to the pandemic response [57] to demonstrate inclusion and equity in recognition of effort. Despite the positive or negative emotions experienced, research conducted concurrently at the same hospital Trust demonstrated high levels of staff dedication and commitment to their roles with the NHS during the pandemic [25].

### 4.3. The Wellbeing Centres

The notion of feeling valued by their employer was central to participants in our study. Despite the pandemic impacts, we found that healthcare workers perceived that there had been a rapid progression in wellbeing support from the early stages of the pandemic, and the wellbeing centres and wellbeing buddies implemented in the participating hospital trust [19] provide one example of efforts that have been made to protect the emotional wellbeing of staff. Steps to manage the emotional impact of the pandemic [7,9,10,19,43,58] and deal with social stigma [19] have been incorporated in early interventions for health and care workers to mitigate the psychological impact of COVID-19.

Our study highlights the agility of leadership during the early stages of the pandemic in promoting and protecting staff wellbeing through the establishment of wellbeing centres. The importance of leadership during a crisis is well documented [59]. Our participants alluded to the rapidity of positive actions to support wellbeing in the early stages of the pandemic, alongside a reduction in the traditional concepts of hierarchy and a move towards a more collegiate leadership style. This breaking down of hierarchical barriers in healthcare, team motivation and the security that comes from camaraderie have been identified as the silver linings of the COVID-19 pandemic [60]. Other studies have identified positive aspects of healthcare workers’ daily activities during the COVID-19 pandemic, including solidarity between colleagues, the establishment of wellbeing support structures and feeling valued by society [61]. However, our participants called for longer-term reductions in bureaucracy, and continuation of effective leadership approaches to sustain momentum in wellbeing focus, and innovation beyond the pandemic. The case for NHS investment in workplace health and wellbeing intervention has been established for many years [62,63], with known benefits for individual and organizational outcomes, such as reductions in sickness absence and improvements in job satisfaction and organizational commitment [64].

It is pertinent that NHS staff in our study did not always feel sanctioned by line managers to engage in self-care, or wellbeing activities at work, including taking work breaks to visit the wellbeing centres. Similar findings have been demonstrated in the context of healthy lifestyle behaviours at work (e.g., physical activity), where job-related barriers to engagement in health behaviours included the structure and nature of the working day (high workload, front line job requirements), workplace culture and norms (resentment from colleagues, no break culture) and organizational concerns (cost of lost time, public perceptions) [65]. Lack of work breaks and an absence of suitable rest areas is particularly common amongst healthcare workers [66,67,68,69], although it is well-established that long hours and consecutive shifts without breaks have negative psychological impacts, and health and safety implications for healthcare workers and patients [23,70]. Within-day work breaks can reduce fatigue and negative emotions [71]. Beyond the restorative effects of breaks, the *quality* of rest areas is also important, with well-designed break areas playing an important role in job satisfaction, work performance and healthcare workers’ perceptions of their potential to positively influence, staff, patient and facility outcomes [72,73].

Although work breaks are often promoted through staff wellbeing initiatives, line managers play a pivotal role in translating wellbeing policies into practice [74]. Studies have highlighted the importance of managers embracing the business case for wellbeing at work and suggested that management relationships can predict employee wellbeing [75]. Our study highlights the challenges staff experience in taking breaks when they are not advocated or encouraged by line managers, and this emphasizes a negative culture around self-care in healthcare services that needs to be addressed. As argued by Edmundson [76], breaks are integral not only to personal wellbeing, but also to patient safety, and workforce sustainability, and going without a break should not be ‘flaunted as a badge of honour’. Work breaks are essential to safety [77]. Guidance on work breaks (and their role in preventing and managing stress and fatigue) could be provided to all staff and line managers, and this is already freely available (e.g., digital package to support the psychological wellbeing of health and care workers through COVID-19 [19]). Tips for staff members on how to discuss the issue of work breaks with their employer are also freely available and could be made accessible to staff [77].

There was a consensus that the wellbeing centres and wellbeing buddies were a valued workplace wellbeing initiative at the participating hospital Trust, as reported by employees, wellbeing buddies and operational staff. This aligns directly with a study reporting centre access rates and views of those who did and did not access the centres during the same time period [19]. Yet, our findings suggest a broader need for greater inclusivity and communication surrounding workplace wellbeing efforts in a healthcare environment, to reach groups that may feel marginalized or experience greater barriers to accessing facilities. Our participants believed that the wellbeing needs of staff working night shifts were less well considered than those working day shifts or more regular ‘office hours’. Yet, shift workers are at high risk of general health concerns [78] including COVID-19 risk [79], and experience high levels of mental ill-health [80,81]. In general, it is proposed that the COVID-19 pandemic impacts low-waged workers more severely than all others, putting them at increased risk of adverse mental health outcomes [82]. However, our participants reported that staff from lower-waged roles were less likely to attend the wellbeing centres. This concurs with data reported previously on uptake and reach of wellbeing centres, showing that lower-waged staff, staff with community-facing roles, and manual workers were less likely to visit these rest areas than frontline care staff in professional roles (e.g., nurses and doctors) [19]. Healthcare organizations need to ensure that wellbeing provisions are provided and accessible to staff in all occupational groups, including shift workers and staff in lower paid roles. Further work is needed to explore wellbeing needs in these groups, address barriers to accessing wellbeing services.

### 4.4. Study Considerations

To set the study findings in context, Office for National Statistics (ONS) figures show that between March-April 2020 (around the first surge of COVID-19 in the UK), and looking at the constituent countries of the UK, England had the highest percentage of deaths involving the COVID-19 in hospitals (68.6%), and the most deaths above the five-year average, with 39,020 deaths registered up to 15 May 2020 which is 45.8% above the five-year average [83]. In April 2020, the Midlands (the specific region of the UK in which this study was conducted) had the highest number of people in hospital with COVID-19 outside of London [84]. The Midlands was one of the seven UK regions with the number of COVID-19 deaths registered being higher than the five-year average by July and August 2020 (around the time our interviews were conducted) [85,86]. This demonstrates the scale of the circumstances experienced by healthcare workers at the participating hospital Trust during this time and reflected on during these interviews. Our data is limited to the views of participants recruited during and shortly after the first surge of COVID-19 in the UK. This may under-estimate the impacts of the pandemic on healthcare workers due to the long-lasting nature of the pandemic and the rapid escalation of positive cases in the UK from COVID-19 in a subsequent surge in the Autumn of 2020 [87].

Only one participant described their current work status as redeployed, however, many of the participants were able to share views from this perspective since several had previously been redeployment and had recently resumed their core job role. Although BAME staff had accessed the wellbeing centres and made contact with wellbeing buddies [19], the views of BAME staff are under-represented in this qualitative study due to a lack of response to our study invitation. Minority ethnic groups are commonly under-represented in clinical and health research [88] with calls to action to address this [89]. Future workforce studies need to consider this in recruitment plans and strive to increase diversity in participants samples. Since, it is possible that engagement of communities and more personalized approaches may be needed to increase participation of minority ethnic communities in research, as has been proposed in studies with various patient groups [88,90,91]. Finally, although the term BAME has been used here, future studies may consider alternatives since a poll by the thinktank British Future suggests that BAME is not well understood and may be too broad to describe the varying experiences of people from different backgrounds.

### 4.5. Summary of Key Findings and Recommendations

**Figure 2:**
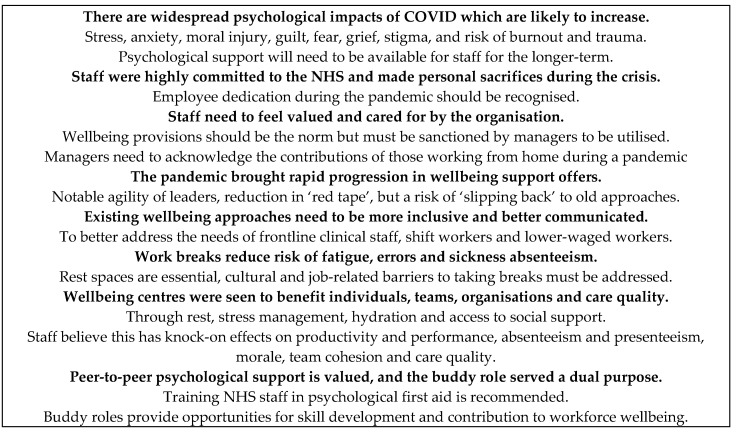
Key findings and interpretation.

Key findings and recommendations have arisen from this work (Figure 2). Having high quality rest spaces is essential for staff wellbeing and these should be made available to staff in all occupational groups and promoted equally amongst them. In particular, healthcare providers need to take actions to ensure an inclusive approach to staff wellbeing, that considers the needs of those staff who feel marginalized. This might include staff that work night shifts, or those working between acute care and community settings. Given the widespread psychological impacts of the pandemic on all staff, the disproportionate impact of COVID-19 on low-paid workers and the paucity of low paid workers using these wellbeing facilities, prioritizing these groups for staff wellbeing initiatives should be a focus. Psychological support (and rest spaces) must be available in the long-term and not just provided as a pandemic response, although efforts need to be made to promote and sanction service use. For this, line manager training and support may be required to encourage prioritization of workforce wellbeing and demonstrate the link between employee wellbeing (including rest, stress and hydration) and morale, patient safety and organizational outcomes. Training staff in psychological first aid has multiple benefits, in terms of providing staff with opportunities to ‘contribute’, facilitating developmental opportunities and operationalizing a mechanism for peer-to-peer support which be a useful adjunct to psychological and support services for healthcare employees. It is important to learn from the agility of NHS Trusts to mobile supportive interventions during the COVID-19 pandemic to ensure this ethos continues as the immediate threat of the pandemic subsides.

## 5. Conclusions

During the COVID-19 pandemic, supported wellbeing centres were set up in UK hospital trusts as an early intervention aimed at mitigating the psychological impact of COVID-19 on healthcare workers. These provided high quality rest spaces with peer-to-peer psychological support from wellbeing buddies trained in psychological first aid. This provision was valued by staff who viewed the centres as critical for the wellbeing of hospital employees during and after the first surge of COVID-19 in the UK. The initiative highlighted a need for equity in provisions, inclusivity and removal of job-related barriers to taking work breaks through managerial role-modelling and advocacy. The model of peer-to-peer support was perceived very positively and afforded many benefits for employees and support workers. Training NHS staff in psychological first aid is recommended as a useful and valued approach to supporting the wellbeing of the NHS workforce during and beyond the COVID-19 pandemic. Overall, high quality rest spaces and access to peer-to-peer psychological support are seen to have broad benefits for individuals, teams, organizations and care quality.

## Figures and Tables

**Table 1:** Sample characteristics for interview participants.

ID ^†^	Gender	Type of Participants	Occupation	Clinical or Non-Clinical Role
101	F	Visitor/Buddy *	Manager	Non-clinical
102	M	Visitor	Ancillary/Maintenance	Non-clinical
103	F	Service */Buddy	Manager	Non-clinical
104	F	Visitor/Buddy *	Manager	Non-clinical
105	F	Visitor/Buddy *	Administrator	Non-clinical
106	F	Visitor/Buddy *	AHP	Clinical
107	M	Visitor/Buddy *	Administrator	Non-clinical
108	F	Service */Buddy	Administrator	Non-clinical
109	F	Service/Buddy *	Administrator	Non-clinical
110	F	Buddy	Nurse	Clinical
115	M	Visitor	Ancillary/Maintenance	Non-clinical
116	M	Visitor	Hospital Volunteer	Non-clinical
118	F	Visitor	Nurse	Clinical
120	M	Visitor	Hospital volunteer	Non-clinical
123	F	Visitor	Nurse	Clinical
125	F	Service/Buddy *	AHP	Clinical
126	F	Buddy	AHP	Clinical
129	F	Visitor	Healthcare Assistant	Clinical
131	F	Visitor	Nurse (redeployed)	Clinical
132	F	Visitor	Nurse	Clinical
135	M	Visitor	Hospital volunteer	Clinical
136	F	Visitor	AHP	Clinical
145	F	Visitor Service */Buddy	Administrator	Non-clinical
147	F	Visitor	AHP	Clinical

* Denotes primary perspective as defined by interviewee (for participants with multiple perspectives). ^†^ Unique identifier assigned by the research team. Visitor: any paid employee as well as bank staff and contracted hospital volunteers working on the study sites during the pandemic; Service: involved in operationalizing the wellbeing centre and/or buddy rotas; Buddy: trained in PFA and worked shift(s) in the wellbeing centres; AHP = Allied Health Professional: occupational therapist, physiotherapist; Redeployed = temporary move to a different job role or return from retirement to clinical practice during the pandemic; Ancillary/maintenance = Estates, maintenance or associated COVID-19 buildings or project work. Hospital volunteer = unpaid staff in contracted and supervised ‘meet and greet’ roles.

## Data Availability

The data presented in this study are available on request from the corresponding author.

## Supplementary Materials

The following are available online at https://www.mdpi.com/article/10.3390/ijerph18073626/s1, File S1: Consolidated criteria for reporting qualitative studies (COREQ): 32-item checklist; File S2: COVID-Well Interview Topic guides, File S3: Analytic framework and codes.
