# Supplementary material for: COVID-Well Study: Qualitative Evaluation of Supported Wellbeing Centres and Psychological First Aid for Healthcare Workers during the COVID-19 Pandemic
Source: Int J Environ Res Public Health. 2021 Mar 31;18(7):3626. doi: 10.3390/ijerph18073626 (PMC8036934; doi:10.3390/ijerph18073626)
Supplement: Supplementary file 1 [file ijerph-18-03626-s001.zip › ijerph-1135653-supplementary/Supplementary file 2_COVIDWell Interview Topic Guides.docx]

**Supplementary file 2.** COVID-Well Interview Topic guides

**COVID-Well Staff Interview topic guide**

Welcome and introduction

1. **If we could start with a little background about you and your current role. What is your current occupation?**

*[prompts: how long have you been a [insert as appropriate]? When did you qualify? Have you always worked in the NHS? How was this impacted on during Covid-19? Did you stay in the same role or were you redeployed during Covis-19 (for how long etc and where)? Did you look after Covid+ patients?)*

Question set

1. **Can you tell me a bit about what was it like working during Covid-19; what were the emotional highs and lows of that time?**

*[prompts: ask for specific stories/ examples]*

1. **As you will be aware from the media Covid-19 affected some members of the community differently with BAME groups more vulnerable than white groups.**
   1. As a member of the BAME community, were you aware of this at the time and did you have any specific concerns or experiences related to this?
   2. (if not BAME group): Were you aware of this at the time and did that affect your experiences in any way (or the experiences of your BAME colleagues?)
2. **What are your views about these Wellbeing Centres being available for staff during the pandemic?**

*[prompts: What do you think of the leadership approach to setting these up? what benefits do you see for staff? Are there any issues with providing these? Is the label appropriate (wellbeing centres)? Are they accessible or are there any problems with access?]*

1. **What was your reason for visiting the Wellbeing Centre?**

*[prompts: were you just passing, or did you plan to attend? Did you attend alone or with a colleague? Were you encouraged to attend by someone else? What was your main purpose for going in? Was this common among your team/peers?*

1. **Did you talk to a Wellbeing Buddy? What are your views about the Wellbeing Buddies (support worker in the room)?**

*[prompts: What are your views on making these support workers available? Were you able to speak to someone if you wanted to? What was the support like?]*

1. **Did you visit to the Wellbeing Centre impact on your psychological wellbeing? In what way?**

*[prompts: how did you feel when you were in the room? And afterwards?]*

1. **Did accessing the Wellbeing Centre have any other particular benefits for you?**

*[prompts: Were you signposted to any resources or services? Did you follow up on any advice you were given by a buddy? Did your visit influence how you felt when back at work or at home?]*

1. **Was there anything that was less helpful or could have been done differently?**

*[prompts: were there any issues in accessing the room, getting there, or when you were in the room? Any problems with getting to speak to someone, or with the person providing advice or the advice or signposting provided?]*

1. **What are your thoughts about the ongoing support needs of yourself or your colleagues in returning to normal duties post-pandemic? How can we best support psychological wellbeing generally?**

*[prompts: are the Wellbeing Centres or similar provisions required in the future? Any other provisions needed for psychological support? What else could be done?*

1. **With regards the Wellbeing Centres, do you have any thoughts or feelings about *how* they are offered and what could be done in the future?**

*[prompts: your thoughts on the current provision – opening times & days, pandemic only or all the time? Anything about the rooms or support workers that should be changed? How can we make this sustainable in the future? What might work?]*

1. **Overall and looking back, what impact (if any) has the Wellbeing Centre had on (i) your overall health and wellbeing, (ii) your views of your employer, and (iii) your commitment to your profession?**

*[prompts: can you sum up the main impacts on you own health and wellbeing?]*

**Close: Is there anything else you would to share with me today?**

**Thanks again for taking the time to speak with me today.**

**COVID-Well Wellbeing Buddy / Service Delivery Interview topic guide**

Welcome and introduction

**1. If we could start with a little background about you and your current role. What is your current occupation?**

*[prompts: how long have you been a [insert as appropriate]? When did you qualify? Have you always worked in the NHS? How was this impacted on during Covid-19? Did you stay in the same role or were you redeployed during Covis-19 (for how long etc and where)? Did you look after Covid+ patients? Or has the Wellbeing Buddy been your main role? (and for how long?)]*

Question set

**2. As you will be aware from the media Covid-19 affected some members of the community differently with BAME groups more vulnerable than white groups.**

- 1. As a member of the BAME community, were you aware of this at the time and did you have any specific concerns or experiences related to this?
  2. (if not BAME group): Were you aware of this at the time and did that affect your experiences in any way (or the experiences of your BAME colleagues?).

**3. What are your views about the Wellbeing Centres being made available for staff during the pandemic?**

*[prompts: What do you think of the leadership approach to setting these up? what benefits do you see for staff? Are there any issues with providing these? Is the label appropriate (wellbeing centres)? Are they accessible or are there any problems with access?]*

**4. What was your reason for taking the role of Wellbeing Buddy in the Wellbeing Centre?**

*[prompts: did you volunteer were you invited, asked or expected to do this? Did you take up the role with other colleagues in your team? Did you know the other buddies?]*

**5. Can you tell me a bit about what was it like being a Wellbeing Buddy in the Wellbeing Centres during Covid-19; what were your experiences of this time?**

*[prompts: ask for specific stories/ examples]*

**6. What are your views about how the Wellbeing Buddies were approached and your support accessed by staff?**

*[prompts: were you approached by many staff for support? Were you able to talk to all of the staff that wanted a chat? What do you think was the main reason that staff attended the centre? Did the process seem to work or not? What went well with regards people accessing a buddy? Were there any challenges for staff in getting to talk to you or another buddy?]*

**6. Did you feel you had the right skills or knowledge for the support and advice you needed to offer in this role?**

*[prompts: Did you receive any training specifically for the buddy role? How useful was the training? Do you have any prior training or work experience that equipped you for this role? Did you feel supported in the role? What are your views on the prior training needed for wellbeing buddies to perform the role effectively? Anything you didn’t know that in hindsight would have helped?]*

**7. What are your views on the impact of the Wellbeing Centres and the Wellbeing Buddies on the psychological wellbeing of staff?**

*[prompts: how did staff seem to feel when they came into the room? How did staff seem to feel when they left? What were the benefits of the Wellbeing buddies for staff? (has this support made a difference to staff wellbeing or not, if so, how?). Did staff tell you about any impacts on their wellbeing from attending the centre or talking to a buddy (yourself or a buddy colleague)]*

**8. How did your conversations generally go? What are your views on how support and signposting was received by staff?**

*[prompts: were staff receptive to your advice? Was the conversation free-flowing? Did you generally provide reassurance, or signposting? Anything else? Do you think that staff valued the support? How do you think they responded? Are you aware of whether staff followed up on advice you gave or suggestions? Did you signpost to any particular resources or services more often than others?*

**9. Did working in the Wellbeing Buddy role have any particular benefits for yourself or your work?**

*[prompts: with regards role, training, skills knowledge etc.]*

**10. Was there anything that went less well, or could have been done differently?**

*[prompts: any issues with choice (or lack of choice) in conducting this role? Were there any issues in people accessing the room, getting there, or when you were in the room? Any adverse events or situations that were difficult to manage? Any problems with getting training or support for yourself?]*

**11. What are your thoughts about the ongoing needs of staff in returning to normal duties post-pandemic? How can we best support staff psychological wellbeing generally?**

*[prompts: are the Wellbeing Centres or similar provisions required in the future? Any other provisions needed for psychological support? What else could be done?*

**12. With regards the Wellbeing Centres, do you have any thoughts or feelings about *how* they are offered and what could be done in the future?**

*[prompts: your thoughts on the current provision – opening times & days, pandemic only or all the time? Anything about the Centres or Wellbeing Buddy role that should be changed? How can we make this sustainable in the future?]*

**14. Overall and looking back, what impact (if any) has the Wellbeing Buddy role had on (i) your overall health and wellbeing (as well as that of the staff you have supported), (ii) your views of your employer, and (iii) your commitment to your profession?**

*[prompts: can you sum up the main impacts on you own health and wellbeing? Can you tell me a bit about your feelings towards your employer?]*

**Close: Is there anything else you would to share with me today?**

**Thanks again for taking the time to speak with me today.**
