# Supplementary material for: COVID-Well Study: Qualitative Evaluation of Supported Wellbeing Centres and Psychological First Aid for Healthcare Workers during the COVID-19 Pandemic
Source: Int J Environ Res Public Health. 2021 Mar 31;18(7):3626. doi: 10.3390/ijerph18073626 (PMC8036934; doi:10.3390/ijerph18073626)
Supplement: Supplementary file 1 [file ijerph-18-03626-s001.zip › ijerph-1135653-supplementary/Supplementary File 1 COREQ_Consolidated criteria for reporting qualitative studies .docx]

**Supplementary file 1.** Consolidated criteria for reporting qualitative studies (COREQ): 32-item checklist

| **Topic and Item No.** | **Guide Questions/Description** | **Response** |
| --- | --- | --- |
| **Domain 1: Research team and reflexivity** | | |
| *Personal Characteristics* | | |
| 1. Interviewer/facilitator | Which author/s conducted the interviews? | Holly Blake, Ben Wood, Steph Knowles, Jennifer Good (early stages of the study only) |
| 2. Credentials | What were the researcher’s credentials? E.g. PhD, MD | Holly Blake: PhD  Ben Wood: BSc  Steph Knowles: MSc  Jennifer Good: MSc |
| 3. Occupation | What was their occupation at the time of the study? | HB: health psychologist/university academic;  SK: health and wellbeing lead  BW: health and wellbeing coordinator  JG: health and wellbeing coordinator |
| 4. Gender | Was the researcher male or female? | 3 female, 1 male |
| 5. Experience and training | What experience or training did the researcher have? | All mixed methods researchers and experienced interviewers  All trained in GCP, research ethics, research integrity and interview skills. |
| *Relationship with participants* | | |
| 6. Relationship established | Was a relationship established prior to study commencement? | Researchers met the participants during recruitment. Rapport was established prior to the study with operational staff. |
| 7. Participant knowledge of the interviewer | What did the participants know about the researcher? e.g. personal goals, reasons for doing the research | Participants knew that HB was a university academic, and that SK, BW, JG were involved in health and wellbeing service delivery.  They knew that the project lead (HB) was not an employee of the participating organisation. |
| 8. Interviewer characteristics | What characteristics were reported about the interviewer/facilitator? e.g. Bias, assumptions, reasons and interests in the research topic | Participants knew that all interviews were interested in the wellbeing of the NHS workforce. |
| **Domain 2: Study design** |  |  |
| *Theoretical framework* | | |
| 9. Methodological orientation and Theory | What methodological orientation was stated to underpin the study? e.g. grounded theory, discourse analysis, ethnography, phenomenology, content analysis | Thematic analysis  Adopted principles of Framework Analysis. |
| Participant selection | | |
| 10. Sampling | How were participants selected? e.g. purposive, convenience, consecutive, snowball | Convenience sample during a set recruitment period. |
| 11. Method of approach | How were participants approached? e.g. face-to-face, telephone, mail, email | Employee mailing lists, social media (NHS Facebook groups and official Twitter sites), and regular departmental mailings and publications. |
| 12. Sample size | How many participants were in the study? | 24 participants took part in qualitative interviews |
| 13. Non-participation | How many people refused to participate or dropped out? Reasons? | 7 agreed to participate but were not available for interview during the study period due to other commitments. No participants actively withdrew. |
| *Setting* | | |
| 14. Setting of data collection | Where was the data collected? e.g. home, clinic, workplace | Data were collected online (video-conferencing platform) |
| 15. Presence of non-participants | Was anyone else present besides the participants and researchers? | Interviews: No. |
| 16. Description of sample | What are the important characteristics of the sample? e.g. demographic data, date | Gender, role (staff, buddy, service), occupational group |
| *Data collection* | | |
| 17. Interview guide | Were questions, prompts, guides provided by the authors? Was it pilot tested? | Question guide is included. It was pilot tested with academic healthcare professionals who were not participants in the study. |
| 18. Repeat interviews | Were repeat interviews carried out? If yes, how many? | No repeat interviews. |
| 19. Audio/visual recording | Did the research use audio or visual recording to collect the data? | Interviews were audio-recorded and saved as mp4 files. |
| 20. Field notes | Were field notes made during and/or after the interview? | Yes. |
| 21. Duration | What was the duration of the interviews or focus group? | Interview length ranged from 22 mins to 63 min and the average duration of interview was 36 min. |
| 22. Data saturation | Was data saturation discussed? | Yes. |
| 23. Transcripts returned | Were transcripts returned to participants for comment and/or correction? | Yes - a sub-sample. |
| **Domain 3: analysis and findings** |  |  |
| *Data analysis* | | |
| 24. Number of data coders | How many data coders coded the data? | Two. |
| 25. Description of the coding tree | Did authors provide a description of the coding tree? | No, however initial coding was informed by the interview guide, and coding was continuously  refined. |
| 26. Derivation of themes | Were themes identified in advance or derived from the data? | These were derived from an a priori framework and also the data (combined deductive-inductive approach). |
| 27. Software | What software, if applicable, was used to manage the data? | None. |
| 28. Participant checking | Did participants provide feedback on the findings? | Yes. |
| *Reporting* | | |
| 29. Quotations presented | Were participant quotations presented to illustrate the themes or findings?  Was each quotation identified? e.g. participant number | Yes. |
| 30. Data and findings consistent | Was there consistency between the data  presented and the findings? | Yes. |
| 31. Clarity of major themes | Were major themes clearly presented in the findings? | Yes. |
| 32. Clarity of minor themes | Is there a description of diverse cases or discussion of minor themes? | Yes. |
