# Supplementary material for: COVID-Well Study: Qualitative Evaluation of Supported Wellbeing Centres and Psychological First Aid for Healthcare Workers during the COVID-19 Pandemic
Source: Int J Environ Res Public Health. 2021 Mar 31;18(7):3626. doi: 10.3390/ijerph18073626 (PMC8036934; doi:10.3390/ijerph18073626)
Supplement: Supplementary file 1 [file ijerph-18-03626-s001.zip › ijerph-1135653-supplementary/Supplementary file 3_Analytic framework and codes.docx]

**Supplementary file 3**. Analytic framework and codes

| Overarching  Theme | Higher-Level Code | Lower-Level Code |
| --- | --- | --- |
| Exposure and job roles | Exposure to COVID-19 | Virus transmission risk  Contacts with family, partners or peers  PPE and training  Duty and responsibility |
|  | Impact of COVID-19 on job role | Increased workload  Changing roles  Care for Non-COVID patients  Technology impacts on work |
| Emotional impacts of COVID-19 | Emotional highs of the pandemic | Making a contribution  Team camaraderie  Employer support for wellbeing |
|  | Emotional lows of the pandemic | Death and grief  Negative emotions  Concerns for the future  Inequity in support |
|  | Ethnicity-specific impacts | Perceived vulnerability  Impact of the media  Conflict and support |
|  | Profession-specific impacts | Integration and co-operation  Perceptions and inclusion  Redistribution of teams |
|  | Return to the ‘new normal’ | Skills for the future  Slipping back to segregation  Delayed psychological impacts |
| The Wellbeing Centres | Centres as a workplace COVID-19 response | Value in staff  Pride in the NHS  Agility of leaders  Investment in staff wellbeing |
|  | Usability and engagement | Reasons for attendance  Work breaks  Barriers to access |
|  | The Wellbeing Buddies | Support for others  Value of the role  Training and support  Workplace culture |
|  | Individual and Team Impacts | Emotional impacts  Time for self-care  Peer support and team relations |
|  | Organisational Impacts | Feeling valued  Team cohesion  Absenteeism and presenteeism  Manager support  Care quality |
|  | Future provisions and support | Wellbeing as the norm  Inclusivity in wellbeing services  Learning from the COVID-19 response |
